# Building causal models for finding actual causes of unmanned aerial vehicle failures

**DOI:** 10.3389/frobt.2024.1123762

**Published:** 2024-02-07

**Authors:** Ehsan Zibaei, Robin Borth

**Affiliations:** Chair of Software and Systems Engineering, TUM School of Computation, Information and Technology, Technical University of Munich, Munich, Germany

**Keywords:** unmanned aerial vehicle, actual causality, natural language processing, causal graph, root cause analysis, automated diagnosis

## Abstract

Finding actual causes of unmanned aerial vehicle (UAV) failures can be split into two main tasks: building causal models and performing actual causality analysis (ACA) over them. While there are available solutions in the literature to perform ACA, building comprehensive causal models is still an open problem. The expensive and time-consuming process of building such models, typically performed manually by domain experts, has hindered the widespread application of causality-based diagnosis solutions in practice. This study proposes a methodology based on natural language processing for automating causal model generation for UAVs. After collecting textual data from online resources, causal keywords are identified in sentences. Next, cause–effect phrases are extracted from sentences based on predefined dependency rules between tokens. Finally, the extracted cause–effect pairs are merged to form a causal graph, which we then use for ACA. To demonstrate the applicability of our framework, we scrape online text resources of Ardupilot, an open-source UAV controller software. Our evaluations using real flight logs show that the generated graphs can successfully be used to find the actual causes of unwanted events. Moreover, our hybrid cause–effect extraction module performs better than a purely deep-learning based tool (i.e., CiRA) by 32% in precision and 25% in recall in our Ardupilot use case.

## 1 Introduction

Small unmanned aerial vehicles (UAVs) are becoming popular in various applications, from medical delivery (Ackerman and Strickland, 2018) to monitoring forests (Torresan et al., 2017). As UAVs achieve higher autonomous capabilities, scenarios in which they may fail also become more complicated. Given the quantity and complexity of software and hardware components in such systems, mere failure detection in a component without causality analysis does not provide actionable explanations.

The case of the open-source UAV controller software, Ardupilot^1^, is a good example. A failure detection program called LogAnalyzer^2^ is developed in this project, which reads flight logs and checks pre-defined rules on each component’s data to determine whether it failed during the flight. Despite LogAnalyzer’s popularity and ease-of-use, many users are still unsatisfied and ask for additional help analyzing their crash logs in discussion fora^3^. This emphasizes that supplying users with only a list of failed components is insufficient. A user needs to know the actual causes of an unwanted event to take corrective action.

**FIGURE 1:**
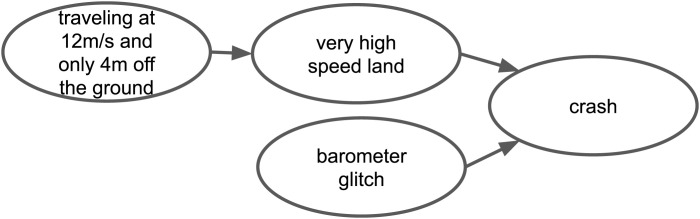
A simplified causal graph built on the basis of two user statements.

Textual causal knowledge from users and developers on online platforms can be useful in this respect. Regarding our example on the Ardupilot project, there are numerous posts in which users share causal knowledge. For example, a user argues in a forum post that “*…traveling at* 12 m*/s and only* 4 m *off the ground, leading to a very high speed land that causes a crash.*”^4^ In another post, another user argues that “*…barometer glitches led to previous crashes.*”^5^ By merging these two pieces of information that have the word *crash* as a common term, we can build a causal graph that consists of four nodes and three directed edges, as shown in Figure 1. This simplified graph suggests that there are two possible scenarios for the crash of an Ardupilot-based UAV.

Suppose all events are binary variables and there is a flight log in which “barometer glitch,” “crash,” and “traveling at 12 m/s and only 4 m off the ground” occurred, whereas “very high speed land” did not occur. In this case, the upper path of the graph could not be a valid causal explanation, because the middle event did not occur. In contrast, the lower path of the graph could be a valid causal explanation. Determining this requires further counterfactual analysis.

We can do a conceptual counterfactual analysis by considering an imaginary world in which all events except “barometer glitch” and “crash” are fixed at their initial state. In this setting, changing the state of “barometer glitch” to not-occurring, will also change the state of the “crash” to not-occurring. In other words, had “barometer glitch” not happened, “crash” would not have happened. Consequently, “barometer glitch” is the actual cause^6^ of “crash”.

Note that in the abovementioned example, two levels of causality are involved: type and actual causality. Type causality describes the generic causality between events by answering questions of the form: “does event A cause event B?” On the other hand, actual causality analysis (ACA) answers questions of the form: “did event A cause event B?” In fact, ACA determines the causality in a specific occasion.

We see type causality analysis as a prerequisite for ACA. Building good causal models for a system typically requires deep domain knowledge and years of experience in working with the system. Such models are typically scarce, even for popular systems such as Ardupilot installed on millions of small UAVs. Thus, the necessity to automate the diagnosis by generating causal models is of utmost importance in developing such systems. The main idea of this study is to learn causal models from natural language texts instead of expensive and time-consuming manual model building.

To realize a natural language processing (NLP)-based solution for generating causal models that can be used in ACA, we identified four major challenges:

**Challenge 1: domain knowledge is scattered among multiple online resources with different structures.** Domain knowledge about technical systems may be found in various resources such as discussion fora and online user manuals with diverse web page structure and semantics. Moreover, discussion texts may contain a considerable amount of noise because the issue is discussed in an informal conversation, where users occasionally thank each other or terminate sentences without proper punctuation marks. This necessitates extensive preprocessing and cleaning.

**Challenge 2: cause–effect phrases may acquire different semantic roles.** Based on the causal verb and the passive or active form of the sentence, cause–effect phrases would fall in with different grammatical structures and can appear in various parts of a sentence. Moreover, there may be multiple cause–effect pairs in each sentence. It is necessary to do a trade-off between the quality and number of extracted cause–effect phrases.

**Challenge 3: extracted phrases should be properly merged and integrated into a single causal graph.** Users may talk about the same concept in different terms. Using a generic thesaurus would not suffice because each technical system’s users have an exclusive terminology that differs from other technical systems. For example, the Ardupilot community uses the words “fault” and “failure” interchangeably, whereas such terms have different meanings in the context of safety standards.

**Challenge 4: Causal models should be adapted to the ACA procedure.** In the ACA literature, nodes of the causal models may be exogenous (i.e., they are instantiated on the basis of a specific system run) or endogenous (i.e., they are instantiated on the basis of their parent node). It is not thoroughly clear how the nodes of a generically built graph should be handled in this respect. Moreover, the generated graphs have to be acyclic to be used in the standard ACA tools.

**FIGURE 2:**
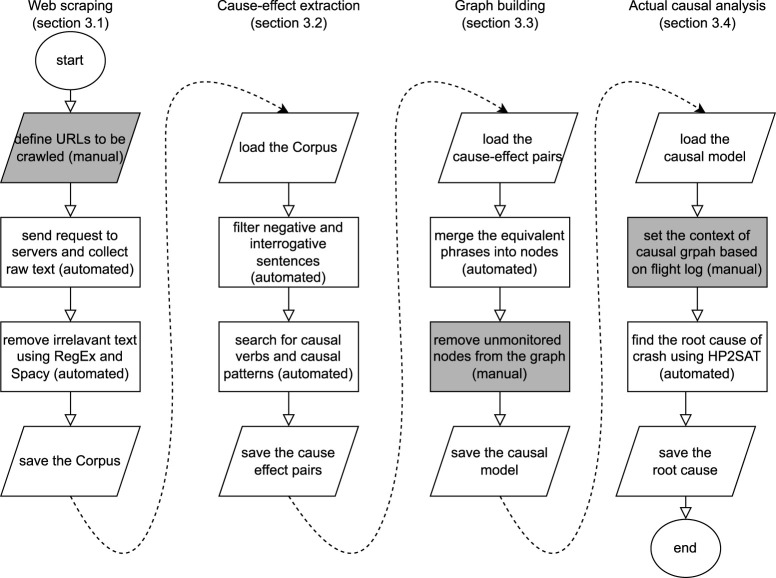
Illustration of the methodology proposed by our framework. Dark cells are manual and light cells are automated processes.

To address these challenges, we propose a technical framework depicted in Figure 2 consisting of four modules: (1) the first module crawls different web page structures and collects a large corpus of text from scattered knowledge over the web, (2) module two finds multi-pair multi-token cause–effect phrases in each sentence, (3) module three merges the discovered phrases into a single causal graph, and, (4) module four finds the actual causes of an unwanted event in a given flight log.

The contributions of this study are the following:

• We propose a generic framework enabling automated collection of textual data and using it for the diagnosis of UAV systems
• Our work addresses the causal model building task, which can highly accelerate the adoption of ACA in the industry
• Our hybrid cause–effect extraction module performs better than a purely machine-learning (ML)-based tool by 32% in precision and 25% in recall in our Ardupilot use case
• We publish a dataset containing four parts: https://doi.org/10.6084/m9.figshare.21711608
  1. ArduCorpus: A corpus of 935K sanitized sentences collected from the Ardupilot UAV user manuals, Discord chat platforms, and discussion fora
  2. ArduCE: A set of 2238 cause–effect pairs that are generated by our extraction module after analyzing ArduCorpus
  3. ArduCrash: Manual analysis of eight real flight logs and their raw data. Although the flight logs are already on the internet, we manually categorize them according to the root cause of the crash and publish the binary events besides the raw time-series data
  4. GroundTruth: A set of 539 sentences as a ground truth in which exact locations of cause–effect pairs are labeled manually

The remainder of this paper is organized as follows: In §2, related studies in web scraping, text processing, and ACA are discussed. Next, our proposed methodology is illustrated in §3. In §4 the primary characteristics of the use cases of this study are introduced. In §5, the proposed methodology is evaluated for its validity and effectiveness. Finally, in §6 conclusion, limitations, and future works are discussed.

## 2 Related work

The gathering of causal knowledge from textual resources has garnered increased attention in the recent years. There are a few end-to-end solutions, such as Ahne et al. (2022) and Maisonnave et al. (2022), proposing frameworks that can sift through large amounts of textual data and detect implicit and explicit causal structures within them. These frameworks could not be used in our context, because Ahne et al. (2022) is customized for a medical domain and Maisonnave et al. (2022) is designed to detect causality between time series instead of binary variables. To generate causal graphs for our specific context, which is the diagnosis of UAVs, we identified four major challenges and discuss existing works in each area in the following paragraphs.

### 2.1 Web scraping

Web scraping transforms unstructured data from one or many websites into unified structured data. A broad range of applications from gray literature search (Haddaway, 2015) to scraping hematologic patients’ information during the SARS-CoV2 Pandemic (Melchor et al., 2020) to gathering social media information (Rajput and Ahmed, 2019) has increased the demand for versatile scraping techniques.

In practice, three main approaches for implementing a web data scraper are available: using desktop-based software, building a web data scraper on the basis of generic libraries, and utilizing existing frameworks. End-to-end software packages such as Fminer^7^ are typically inflexible in working with various website formats and data structures. Combining general-purpose libraries, as proposed in Mitchell (2018), is not a robust solution, because occasional changes in the web resources would cause the scraping to fail. Scraping frameworks provide a balanced solution by gathering various components under a unified architecture. One of the most well-known frameworks is Scrapy^8^, which has been successfully used in big data applications (Chaulagain et al., 2017; Landers et al., 2016). Another challenge are dynamic web pages that may require JavaScript scripts to be executed to extract content. This can be performed by Selenium^9^, developed originally for website testing, which is directly available in the Scrapy framework. Given Scrapy’s mature technology and versatility, we build our scraping module on top of it.

### 2.2 Cause–effect extraction

Detecting cause–effect phrases in natural language sentences has been extensively studied in the literature. See (Yang et al., 2021) for a survey of extraction techniques and examples of detecting implicit and explicit causal relations. We identified two major objectives in the literature for extracting cause–effect phrases from a given set of sentences.

The first objective is to determine whether a given pair of phrases is causal according to a text. Doan et al. (2019) surveys Twitter posts for answering the question, “Does stress cause insomnia?” by searching for several causal patterns, including an active form, a passive form, and an active form with a proposition. Our work evaluates combinations of their rules in the context of UAVs. Sharp et al. (2016) proposed to trace back outgoing dependency links from root tokens to expand the detected token into a phrase. Their method can detect multi-token cause–effect phrases instead of single-token phrases. Similarly, Sorgente et al. (2013) proposed an approach to finding multiple pairs in a sentence based on conjunction rules. Our work combines the methods of Sharp et al. (2016) and Sorgente et al. (2013) to achieve multi-pair multi-token cause–effect detection in each sentence.

The second objective is recently reported in a series of works (Frattini et al., 2020; Fischbach et al., 2021) that aim to collect previously-unknown cause–effect phrases in natural language texts. Their tool, CiRA, works based on a deep neural network trained on a corpus of 8,430 sentences. Although most of the training sentences are in the form of if–then statements, CiRA, is claimed to be able to extract cause-effect phrases from any type of sentence. Hence, we include it as a baseline in our evaluations.

### 2.3 Building causal graphs

Causal knowledge is typically represented through a directed graph, which encodes events as nodes and causal relationships as edges (Pearl, 1998). This representation strongly resembles how the human mind perceives causal relationships in complex systems. A few studies (Zibaei et al., 2018; Ibrahim et al., 2019a) investigated converting standard fault and attack trees of UAVs into causal graphs. These studies assume that fault and attack trees are initially available, which does not address the core problem fundamentally.

The causal graphs we seek to build in the first step are a type of directed graphs that should be acyclic (Morgan and Winship, 2014). Another issue that arises is the level of abstraction of the discovered phrases. Our extraction module outputs a list of phrases that need to be integrated at a single graph. The baseline approach is to merge the exactly same phrases into single nodes. Another technique is to merge the phrases on the basis of their syntactical and semantical aspects (Khurana et al., 2022). We study how different merging techniques affect the structure of the generated graphs.

A more advanced approach is to train a word embedding (Kutuzov et al., 2017) on the basis of a specific context and use it to match similar phrases. In that case, for example, “GPS glitch” and “GPS disconnection” will have a very small vector distance and could be merged. For example, Hassanzadeh et al. (2019a) employed neural networks to effectively capture semantic relations across phrases. Building such customized word embeddings is an open problem and out of the scope of this study.

### 2.4 ACA

ACA is an essential task in any diagnostic procedure. Various languages, definitions and benchmarks are surveyed in Kueffner (2021) for actual causality. Among them, Halpern and Pearl (HP)’s definition in Halpern (2015) is the most popular formalism, which works on the basis of causal graphs. This approach is built on top of three rules, which we discuss in §3.4. Ibrahim et al. (2019b) automated the process of checking these conditions through SAT solving and contributed a tool, HP2SAT in Ibrahim and Pretschner (2020), to perform the reasoning. We use HP2SAT to perform ACA and indirectly evaluate the validity of the generated causal graphs.

## 3 Proposed approach

This section illustrates each of the four modules in our proposed framework depicted in Figure 2. The framework is generic in the sense that it can be applied to any UAV platform with minimal changes, given that the textual domain knowledge and runtime logs are available. Our use case are UAVs equipped with Ardupilot as the autopilot system.

### 3.1 Scraping web resources

Web page scraping consists of two tasks: defining the desired links, i.e., web crawling, and parsing the response of the server. Based on Scrapy’s Spider classes^10^, we define the desired links and the depths to follow in addition to the customized parsing functionality. Some web pages such as those in discussion fora may load dynamically, which requires user interaction. We use Selenium^11^ to automate web browser interaction and gather content by applying manually defined XPaths and CSS selectors to user statements.

After extracting data from different sources, the output may still be poorly formatted because of errant punctuations, inconsistent capitalization, line breaks, and misspellings. We detect sentence boundaries using Spacy’s^12^ pre-trained NLP models. Data from discussion fora or chat platforms require additional cleaning due to duplicate posts mentioning each other and broken sentences due to the presence of graphical objects in texts, which we perform by applying regular expression patterns.

### 3.2 Extracting cause–effect pairs

Extraction is the core task in our framework and highly affects the downstream diagnosis results. We propose a hybrid extraction procedure that consists of data-driven and rule-based constituents. Decomposing the gathered sentences into tagged tokens is performed using pre-trained NLP models, while identifying the cause and effect phrases in each sentence is realized by using a rule-based technique.

As a preliminary step, we drop three types of sentences: (1) interrogative sentences, (2) sentences that do not contain a verb, object, or subject, and (3) sentences that contain at least one negated word^13^. We follow the procedure proposed in Girju and Moldovan (2002) for identifying the potentially causal sentences. They distinguish between low and high ambiguity causal verbs. Hence, we search for the sentences that have at least one item of a list of 25 low-ambiguity causal keywords^14^. This filtering process outputs potentially causal statements.

#### 3.2.1 Detecting root tokens of cause–effect pairs

Stanford dependencies (DEP) tags introduced in De Marneffe and Manning (2008) are very useful for localizing the root token of cause–effect phrases. DEP tags provide a simple description of grammatical relationships in a sentence. We use such dependencies to define causal patterns to be detected in sentences. Spacy’s 4-step pipeline can be used to assign DEP tags to tokens:

1. **Tokenizer** splits a text into simple segments, called tokens. These tokens could be words, punctuations, combinations of abbreviations, and so on.
2. **Tagger** assigns POS (parts of speech) tags to the tokens to specify their grammatical roles in a sentence. Major classes in standard POS tagging are adjective (ADJ), noun (NOUN), proper noun (PROPN), and verb (VERB).
3. **Lemmatizer** groups different inflected forms into a single word. This component of the pipeline is used in the graph-building step.
4. **Parser** adds DEP tags to the tokens. The tags used within our detection patterns are “nsubj,” “nsubjpass,” “dobj,” “prep,” “pobj,” and “agent.” The meanings of major DEP tags used by us are shown in Table 1. A more complete list can be found in Choi and Palmer (2012).

**FIGURE 3:**
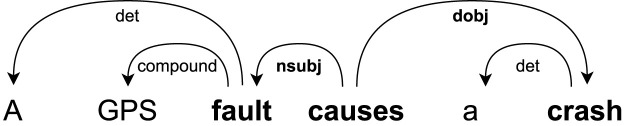
DEP pattern for a causal sentence in active form.

Once the tokens of each sentence are tagged, we use the three patterns proposed in Doan et al. (2019) to detect cause–effect phrases:

**The first pattern** focuses on the form in which the cause is the subject and the effect is the object. An example of this pattern is shown in Figure 3, where the arcs represent the dependencies between tokens. In this sentence, “causes” is the causal keyword, “fault” is the cause root–token, and “crash” is the effect root–token.

**TABLE 1:** Meaning of DEP tags.

Tag	Meaning
nsubj	Nominal subject of the clause
nsubjpass	Nominal subject of the passive clause
dobj	Accusative object of the verb
pobj	Root of a noun phrase following the preposition
prep	Preposition that follows a verb
conj	the relation between two elements connected by a coordinating conjunction, such as “and” and “or”
agent	Complementary part of a passive verb that sets the stage for introducing the performer of the action

**FIGURE 4:**
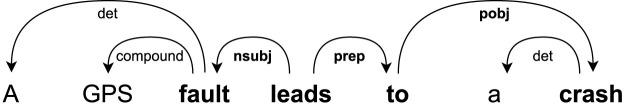
DEP pattern for a causal sentence with preposition.

**The second pattern** is designed to detect causal mentions in which the causal keyword is followed by a preposition such as “in,” “of,” or “to.” For this pattern, the cause is linked to the verb with “nsubj,” whereas the effect is “pobj” linked to the causal verb via preposition: “prep.” The output of detecting this pattern for the sentence “A GPS fault leads to a crash.” is depicted in Figure 4.

**FIGURE 5:**
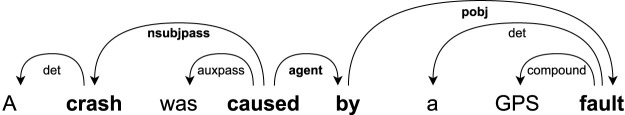
DEP pattern for a causal sentence in passive form.

**The third pattern** is proposed for detecting passive causal statements such as “A crash was caused by a GPS fault.” In this pattern, “by” is tagged as “agent” and accompanies the causal keyword. The cause is “pobj,” which is linked to “agent.” The effect is “nsubjpass,” directly linked to the causal keyword. An example of this pattern is depicted in Figure 5.

#### 3.2.2 Detecting multi-token cases

Considering the example: “A GPS fault causes a crash,” it is clear that “fault” → “crash” is not sufficiently informative for diagnostic applications. A better extraction would return “GPS fault” → “crash.”^15^ Similar to Sharp et al. (2016), to expand the root token into a phrase, we collect the tokens, which are connected to the root token via specific DEP dependency tags.^16^

#### 3.2.3 Detecting multi-pair cases

Now consider an even more complex example: “An empty and defect battery, a GPS fault or a defect motor cause a crash.” By considering our collected sentences, we realized that in a conversational text, “and” and “or” are loosely used without implying a logical meaning. Given that speakers use “and” and “or” interchangeably to refer to a set of elements, the safest approach is to treat all pairs as disjunctions. Hence, the above sentence contains four cause–effect pairs to be detected:

• empty battery causes a crash
• defect battery causes a crash
• GPS fault causes a crash
• defect motor causes a crash

To detect multiple causal pairs in one sentence, we rely on “conj” DEP tag. Similar to Sorgente et al. (2013), we take tokens connected by “conj” for separate causes of the effect. If there are more than one causes and more than one effects, we extract all combinations of the detected causes and effects.

### 3.3 Building causal graphs

Causal graphs have two main aspects: structural and functional forms. The structural form is a graphical representation that determines which nodes causally influence each other. The functional form specifies to what extent and under which functionality each node influences its child node. Knowing the structural form is a sufficient starting point for diagnostic applications. Hence, finding the extent to which each parent node influences its child nodes is considered out of the scope of this study. Moreover, we assume that graph nodes are binary variables that either occur or not occur in the course of the UAV flight.

To form the causal graph structure, we first initialize one node for each distinct member of the extracted cause–effect pairs. Then, we assign a directed edge between nodes that are in the same cause–effect pair. When a phrase is the effect in two distinct cause–effect pairs, we consider the relationship as a disjunction of the two causes. This baseline merging technique enables us to form an inclusive graph containing all collected cause–effect phrases.

It is also possible to equate similar phrases to achieve a more compact graph. The challenge to be addressed here is that humans use different terms to talk about the same concepts. This issue is highly remarkable in Ardupilot, which lacks a standardized taxonomy of the system-related concepts. Furthermore, the community of this open-source project includes people from all over the world with varying levels of English proficiency, which increases the diversity of terms used in the discussions. In addition to the baseline, we consider three merging techniques on the basis of the preprocessed tokens:

• In **lemma-based merging**, we equate phrases that have the same lemma. In this way, for example, “worse GPS connection” will be represented by “bad GPS connection” node in the graph, because “bad” is the basic morphologic form of “worse”.
• In **POS-based merging**, phrases are set equal when their tokens with “NOUN,” “PROPN,” and “VERB” POS tags are the same. In this technique, we only keep tokens with the mentioned POS tags. This technique merges the two abovementioned phrases into one “GPS connection” node.
• In **root-based merging**, we equate two phrases when the root token of them, which is assigned on the basis of DEP tags, are the same. If, in our example, GPS is the root token for both phrases, we would merge them to the node “GPS.”

Two issues need to be addressed for preparing the causal graphs to be used in HP2SAT. First, the raw graphs may contain cycles, however, HP2SAT is only compatible with acyclic graphs. We use the fact that in our application, there should always be a to-be-diagnosed unwanted event. Hence, we calculate all shortest paths between the unwanted event and its ancestor nodes. Then, we drop the ones that are subsets of other detected paths. This gives us a connected and acyclic graph that can be directly used by HP2SAT library.

Second, while the occurrence or non-occurrence of the root events in the causal graph (i.e., exogenous variables) should be specified for the HP2SAT tool, some of them may have not been monitored by the UAV logging mechanism. For practical reasons, we drop the paths that do not start from a monitored event. Note that if the set of monitored events is small, then this step would significantly reduce the size of the graph. Similar to other diagnostic solutions, our ACA module depends on the inclusiveness of the logging mechanism to produce comprehensive results.

### 3.4 Using causal graphs

We follow HP’s definition in Halpern (2015) to find the actual causes of UAV failures. When sufficient evidence (e.g., a run-time log) and a causal graph are available, HP’s definition can determine whether an event (or a conjunctional set of events) is the actual cause of an unwanted event. According to HP’s definition, if the following three conditions hold for the events A and B, then A is the actual cause of B:

1. The occurrence of both A and B is recorded in the logs.
2. Two counterfactual conditions hold:
  • If, in a hypothetical world (i.e., our causal graph), A is flicked off, B will also flick off.
  • If, in a hypothetical world (i.e., our causal graph), any other node rather than A and B flicks off, B does not flick off.
3. If A is a conjunctional set of nodes, it is minimal.

The first condition makes sure that the events in question really occurred in the logs, otherwise there is no need for considering them. The second condition, which has its roots in the philosophical views on actual causality, checks counterfactual scenarios. The third condition is proposed to comply with the principle of parsimony in generating minimal diagnoses. We use the “inference” functionality of HP2SAT (Ibrahim and Pretschner, 2020) to find all actual causes of specific events in specific flight logs.

For diagnosing a specific flight log, the occurred events in it should be specified as the “context” in HP2SAT. For the sake of comprehensibility, we consider an event as “occurred” in our analysis, if it occurs at least once in the course of the flight. An alternative approach is to enumerate the instances of the same event and consider them separately in the analysis. This assumption is not restrictive in the context of UAVs, because the length of flights are typically less than 10 min and most events such as *take-off* and *crash* occur only once.

## 4 Use cases and the ground truth

### 4.1 Internal use case

In the past decade, Ardupilot^17^, a successful open-source autopilot project, has emerged, enabling small robotic systems to perform autonomous missions. Because of its robustness and versatile functionalities, it has been used in numerous scientific and commercial applications (Baldi et al., 2022; Baidya et al., 2018; Luo et al., 2019). Given the large number of developers, 640 as of Autumn 2022, it has grown to cover different vehicle platforms, including rovers, boats, and different types of aerial robots. The project consisting of 700 k lines of code is now mature but complex.

Another interesting aspect of Ardupilot is that its developers and users use various discussion platforms, which are also accessible to public. Users ask questions in discussion fora. Developers discuss existing bugs and new features in Discord chats. In addition, there are user manuals written by experienced developers who possess valuable knowledge about the system.

Ardupilot has also a relatively better logging system than other open-source autopilot platforms, such as PX4^18^ and Paparazzi^19^, in that, abstracted events are monitored and recorded at runtime in addition to raw sensor outputs. This enables us to effortlessly map the mentioned events by users and developers to flight log events. For example, *take-off*, and *GPS failsafe* are already recorded as binary events in the flight logs. In contrast, PX4 flight logs only contain time-series data necessitating an abstraction process to detect events in the time-series data and then map them to the concepts that users and developers talk about.

### 4.2 External use case

Strategic Foresight Analysis Report (NATO-SFA)^20^ is an annual publication by NATO which summarizes “trends that will shape the future security environment and possible implications for the Alliance.” It also includes generic geopolitical findings and prognoses about the world in textual form. Hassanzadeh et al. (2019b) published a dataset that contains cause–effect phrases implied by this document. We manually complemented the cause–effect phrases with random causal verbs to build complete sentences. Such a Synthesized dataset is of interest to us for two reasons: first, the cause–effect terms in NATO-SFA are used in the literature as a benchmark to evaluate the accuracy of binary causal classifiers (Kayesh et al., 2020; Hassanzadeh et al., 2019a); Second, documentations of commercial UAVs are more likely to be concise and straightforward compared to open-source UAVs. Hence, having a concise text, similar to our synthesized NATO-SFA dataset, gives us a rough estimation of how accurate our cause–effect extraction module would work, if we had a very well-written descriptive text for UAVs.

### 4.3 Building GroundTruth dataset

The input to the extraction module are raw sentences and the output are cause-effect phrases. To evaluate the performance of the extraction module, we need a labeled dataset as the ground truth. The labeled dataset should specify which tokens in each sentence constitute the cause and effect phrases.

To our knowledge, there is no such labeled dataset in the literature that specifies exactly where cause and effect phrases lie in a sentence. Thus, we randomly selected 439 sentences from ArduCorpus and 100 sentences from NATO-SFA document. Two authors of this paper annotated the cause–effect phrases. The rate of agreement for the two annotators is 76%, which is not very high, because labeling the cause–effect tokens is such a delicate task that even domain experts may interpret natural language statements differently. In any case, the agreement rate of 76% indicates that the annotations were not erratic. For measuring the performance of our techniques, we take the mean performance among the two ground truth datasets.

## 5 Results

In this section, we (1) review scraping and data cleaning results, (2) assess how accurately cause–effect phrases are extracted from the text, (3) determine how each merging technique affects the structural characteristics of the generated graphs, and (4) investigate the validity of diagnoses generated by our ACA module. In particular, we answer the following research questions:

**RQ**_**1**_: How effective are the pattern-based rules in identifying the cause–effect pairs?
**RQ**_**2**_: How does our hybrid extraction module perform compared to CiRA?
**RQ**_**3**_: How much does cause–effect extraction improve when larger NLP models are used in the pipeline?
**RQ**_**4**_: To what extent does the conciseness of the text affect the extraction performance?
**RQ**_**5**_: Which merging technique outputs better causal graphs in terms of the validity of the diagnoses?

### 5.1 Web scraping results

By applying our scraping module, we collected approximately 1.8M pieces of text from Ardupilot online resources, with 1.3M coming from discussion fora, 380K from user manuals, and 120K from Discord chats. After preprocessing and cleansing the raw pieces of text, we reduced the size of the corpus to 935K sentences. We publish this dataset as ArduCorpus.

### 5.2 Extraction results

#### 5.2.1 Finding causal keywords

The extraction procedure begins with finding the causal keywords. From 935K sentences in ArduCorpus, only around 40K contained one or more instances of our 25 causal keywords. In particular, we found that “link,” “cause,” “result,” “lead,” and “trigger” constitute around 75% of the detected keywords in ArduCorpus.

**FIGURE 6:**
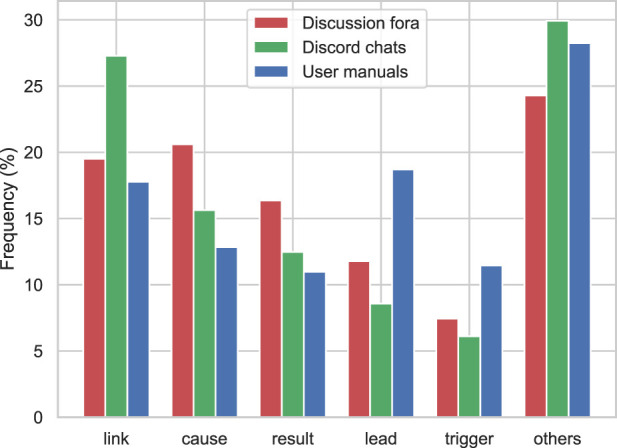
Distribution of causal keywords in ArduCorpus.

According to Figure 6, which depicts the distribution of causal keywords, more formal and technical keywords such as “lead” and “trigger” are used in user manuals, whereas different causal keywords such as “cause” and “result” are prevalent in discussion fora. Moreover, the “link” keyword is most common in Discord chats. One reason is that Discord chats are mainly used by developers who usually mention development issues in the form of links in their arguments. Nevertheless, this type of usage for the “link” keyword does not match any of the three patterns described in §3.3 and our causal extraction algorithm disregards such usages of it.

#### 5.2.2 Extracting cause–effect phrases

Because our framework allows for the extraction of multiple tokens and multiple pairs in every sentence, we cannot directly use the performance measures from other studies that only extract single tokens and single cause–effect pairs. To resolve this issue, we employ the Jaccard index and harmonic mean, similar to the object detection practice in the image processing field, where several objects of different sizes have to be detected in each image. The evaluation procedure is similar to the evaluation of object detection tasks with one difference: objects are cause–effect pairs in this case.

We define the token detection rate (TDR) as the Jaccard index of the detected tokens in Eq. 1:

$$TDR=\frac{\left|detected\text{\_}tokens\cap correct\text{\_}tokens\right|}{\left|detected\text{\_}tokens\cup correct\text{\_}tokens\right|}$$ (1)

Correct tokens are specified by the ground truth explained in 4. For example, if the correct phrase contains three tokens in “bad GPS connection,” and only “GPS connection” is detected by the extraction module, the detection achieves a TDR of 2/3. The denominator in this measure prevents extraction algorithms to exhaustively pick out all tokens for the cause–effect phrases. Next, because each causal pair consists of two phrases (i.e., cause and effect), we need to take the detection rate of both phrases into account. Hence, we define pair detection rate (PDR) in the sentence as the harmonic mean of cause and effect TDRs in Eq. 2:

$$PDR=\frac{2\times TDR_{\mathrm{cause}}TDR_{\mathrm{effect}}}{TDR_{\mathrm{cause}}+TDR_{\mathrm{effect}}}$$ (2)

To determine whether the pair was correctly detected, we convert the continuous PDR into a binary value using pair detection threshold (PDT). If the PDR is above PDT, the pair is considered to be correctly detected, i.e., true positive. If the PDR for a detected pair is below PDT, that pair is considered to be incorrectly detected, i.e., false positive. If a pair is not detected by the algorithm, that is a false negative. We use standard definitions of precision, recall, and Matthews correlation coefficient (i.e., MCC) in Eq. 3:

$$\begin{array}{ll} Precision & =\frac{TP}{TP+FP},\\ Recall & =\frac{TP}{TP+FN},\\ MCC & =\frac{TP*TN-FP*FN}{\sqrt{\left(TP+FP\right)*\left(TP+FN\right)*\left(TN+FP\right)*\left(TN+FN\right)}} \end{array}$$ (3)

The above measures allow for the evaluation of causal extraction algorithms by taking the location of multiple cause–effect phrases into account.

Note that choosing an appropriate PDT value should be based on the requirements. If accurate localization of cause–effect phrases is important, a high threshold should be chosen. In the literature, PDT is usually treated superficially and the smallest overlap between the detected phrase and correct phrase is taken as a true positive. Because there is no consensus in the literature on choosing the PDT, we characterize the performance of the extraction module based on this parameter. Moreover, to ensure the generalizability and robustness of our results, we split the GroundTruth dataset into 5 folds and reported the mean of the precision, recall and MCC among these 5 folds as the final value. This approach ensures that the algorithm is robust and performs equally well on all subsets of the original dataset.

**FIGURE 7:**
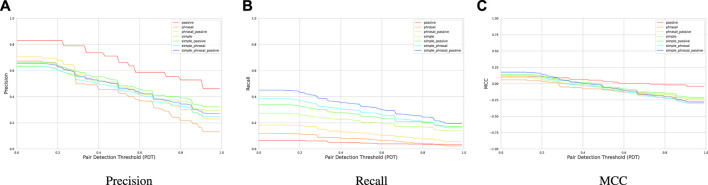
**(A)** Precision, **(B)** Recall, and **(C)** MCC of various combinations of patterns based on the Ardupilot sentences of the GroundTruth.

In Figure 7, precision, recall, and MCC for all seven combinations of the three causal patterns from §3.2 is plotted. For all combinations, as the PDT increases precision, recall, and MCC degrade. The *passive* pattern achieves the highest average precision of 67%, however, it has the lowest average recall of 5%. The *simple-phrasal-passive* combination, achieves a moderate average precision of 48% while having the highest average recall of 33%. MCC does not seem to be influenced by the number of rules.

Our extraction module shows the feasibility of a hybrid cause–effect extraction technique that uses NLP to preprocess the sentences and then extract the cause–effect phrases based on manually-defined rules. Although the performance of our extraction module is not high, these results demonstrate that employing more versatile and accurate rules may increase the precision and recall, which will consequently improve the downstream diagnosis results.

**RQ1**: We find that composing several patterns generally increases the recall in the cause–effect extraction task. The *simple–phrasal–passive* pattern achieves the highest average recall (33%) while maintaining a moderate average precision (48%).

**FIGURE 8:**
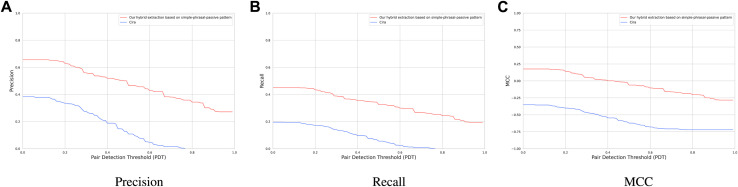
**(A)** Precision, **(B)** Recall, and **(C)** MCC of our hybrid extraction module vs. CiRA.

Figure 8 compares the performance of our rule-based extraction technique to CiRA on the Ardupilot sentences of the GroundTruth dataset. CiRA achieves an average precision and recall of 16% and 8% respectively. The average precision and recall of our hybrid extraction module are 32% and 25% higher than CiRA. The reason for the low performance of CiRA on the GroundTruth dataset is that, although CiRA is the most similar tool in the literature to our cause-effect extraction module, it is not mainly designed for detecting generic causal statements. Given that requirements engineering documents are typically in the form of if–then statements, it is mainly trained it to detect if–then statements. The authors of CiRA assume that if–then statements are causal. CiRA exclusively regards if-then structures as causal, whereas we interpret these structures as non-causal. Consequently, CiRA achieves a mean MCC of −57% for the Ardupilot sentences of the GroundTruth dataset. Nevertheless, CiRA is based on a state-of-the-art data-driven approach which can be significantly improved, if it is trained to detect other causal structures.

**RQ2**: We find that our hybrid extraction module performs on average 32% and 25% higher than CiRA in precision and recall.

**FIGURE 9:**
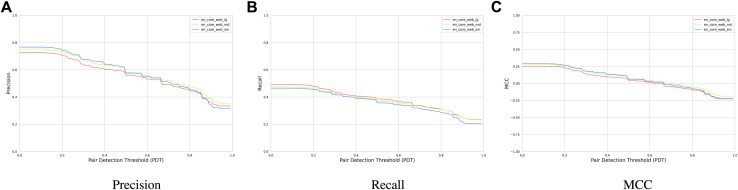
**(A)** Precision, **(B)** Recall, and **(C)** MCC of our hybrid extraction module using three NLP core models.

Next, we investigate the extraction performance in terms of the enormity of NLP core models. Figure 9 depicts how precision, recall, and MCC differ when using small (12 MB), medium (40 MB), and large (560 MB) NLP models from Spacy. The plot shows that the performance is not influenced significantly when different NLP models are used. Hence, using larger models that are trained on the generic text rather than a domain-specific text does not improve the extraction performance.

**RQ3**: We find that using larger generic NLP models does not significantly improve the cause–effect extraction performance.

**FIGURE 10:**
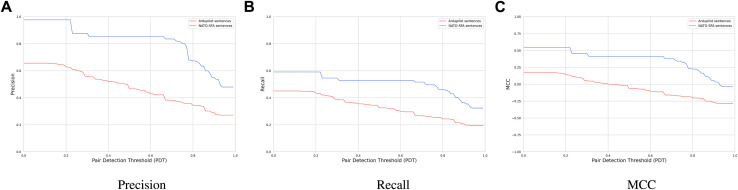
**(A)** Precision, **(B)** Recall, and **(C)** MCC of our hybrid extraction module on the sentences from NATO-SFA and Ardupilot.

Figure 10 depicts precision, recall, and MCC of the extraction module on the two subsections of the GroundTruth dataset. Our extraction module achieves an average precision and recall of 82% and 51% respectively in the NATO-SFA sentences, which is higher than its performance on the Ardupilot sentences of the ground truth. This stems from the fact that our NATO-SFA sentences are crafted in a structured manner, which makes it easier for the extraction algorithm to detect cause–effect phrases. This observation emphasizes the fact that in addition to other engineering tasks in the development process, diagnostic tasks would also benefit from a structured documentation in the long-term.

**RQ4**: We find that our hybrid extraction module achieves on average 34% higher precision and 18% higher recall on NATO-SFA sentences. This can be attributed to the conciseness of the sentences in NATO-SFA compared to the sentences from Ardupilot.

The output of the extraction module are 3558 distinct phrases that constitute the cause–effect pairs. We publish these cause–effect pairs as ArduCE dataset.

### 5.3 Graph building results

The merging techniques introduced in §3.3 aim to unify similar nodes in the graph. In this subsection, we characterize them in terms of affecting the graph structure properties. Equation 4 specifies how to measure the density of the generated graphs as defined in Coleman and Moré (1983):

Density=|Edges|2|Nodes|2=|Edges||Nodes||Nodes|−1 (4)

The extraction step resulted in 3558 distinct phrases. Thus, our baseline merging technique builds a graph that consists of 3558 nodes. Table 2 presents the node count, edge count, the number of tokens per node, and the density of the graph for each merging technique. Root-based increases the density more than other merging techniques. This can be attributed to the functionality of root-based merging technique in reducing the phrases into single tokens. We also observe that lemma-based merging actually increases the number of tokens per node, whereas POS-based and root-based merging reduce it compared to the baseline. This can be explained by considering the fact that POS-based and root-based merging techniques filter tokens on the basis of their semantic roles. In contrast, lemma-based merging technique transforms the phrases to equivalent phrases without dropping tokens.

**TABLE 2:** Characteristics of the generated graphs from different merging techniques.

Merging technique	Nodes	Edges	Tokens per node	Density
Baseline	3558	2503	2.64	0.00020
Lemma-based	3376	2490	2.70	0.00022
POS-based	2747	2422	1.97	0.00032
Root-based	1271	1970	1.00	0.00122

Note that validating the generated causal graphs requires a concrete ground truth that typically does not exist in practice. Even the most adept users do not have a comprehensive understanding of the entire UAV system and its component interactions. The only suggested method for validating a causal relationship, according to the causal inference literature (Gentzel et al., 2019), is random experimentation. Performing random experimentation for validating each edge of a large graph is impractical. Hence, we indirectly assess the causal graphs by applying them to UAV diagnosis tasks in § 5.4, where we analyze the validity of the diagnoses.

### 5.4 ACA results

Wild et al. (2016) surveyed official reports of 152 civil UAV flight incidents that occurred between 2006 and 2015. The reports address the managers and regulators, not the technical stakeholders and hence, are not deeply technical. Nevertheless, Wild’s findings on the categorization of the causes of UAV failures is relevant for our application. They argue that in contrast to manned aircraft in which, failures typically have roots in the operator error, failures of UAVs are due to the functional problems in the system. They conclude that the three most frequent categories of UAV failure scenarios are: (1) loss of control in-flight, (2) events during takeoff and in cruise, and (3) equipment problems.

Given that the manual validation of the diagnosis results is a very time-consuming task due to the large size and dimensions of the flight logs, only eight flight instances belonging to above mentioned categories were analyzed by ACA. Nevertheless, higher heterogeneity is a necessary factor in evaluating any diagnosis framework and hence, extensive study on numerous flight logs is needed to draw conclusions on the framework’s effectiveness.

**FIGURE 11:**
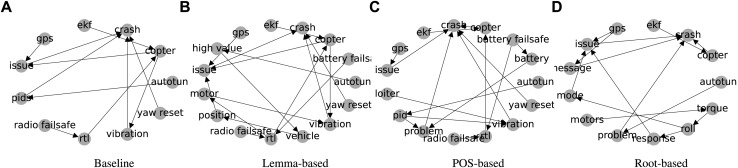
Depiction of **(A)** Baseline, **(B)** Lemma-based, **(C)** POS-based, and **(D)** root-based graphs after dropping the nodes that are either not monitored or do not have a path to the Crash event.

One of the most important unwanted events in Ardupilot flight logs is the *crash* event. The monitoring mechanism for the *crash* event runs continuously during the flight, and once the sensor readings match the specified criteria^21^, an instance of the *crash* event is recorded in the flight logs. The *crash* event (as a class, not as an instance) also exists in our generated graphs. The cuts of the generated graphs containing the *crash* event and its monitored ancestors (as specified in §3.4) are depicted in Figure 11.

To diagnose the crash instances in the ArduCrash flight logs, we import the four generated causal graphs into HP2SAT. Next, we set the “context” according to the monitored events in each flight. Finally, we run the “inference” functionality of HP2SAT to compute the actual causes of the *crash* event in each flight log.

#### 5.4.1 Category 1: loss of control in-flight

**FIGURE 12:**
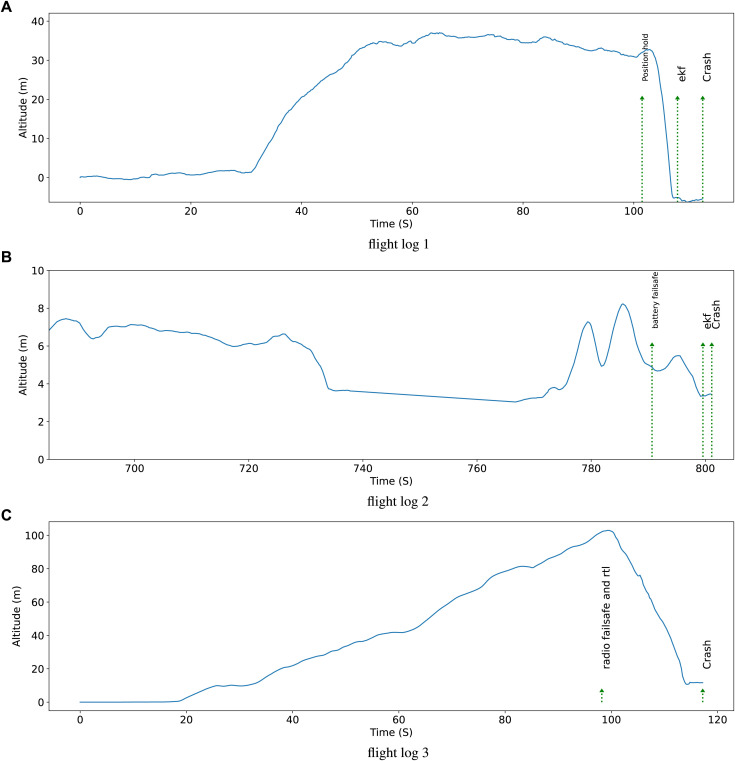
Altitude signal and important events during flight logs.

Flight logs 1 to 3 belong to this category. Due to space constraints, we provide a summary of the diagnoses in the first column of Table 3, while elaborating only on flight log 1 in detail here. The altitude signal of the UAV during flight log 1 in addition to the important events are depicted in Figure 12. In total the UAV was airborne for 70 s. In the last seconds before crash, the UAV was performing a fast maneuver to change its direction. This maneuver is very demanding even for high performance multicopters and typically results in the loss of control. Moreover, the inadequate controller parameters contributed to the destabilization and crashing of the UAV.

**TABLE 3:** Actual causes of the *crash* event according to HP2SAT using the generated graphs.

GraphLog#	1,2,3 (Category 1)	4,5 (Category 2)	6,7,8 (Category 3)
Baseline	[ekf], [copter]	[copter], [radio failsafe]	[*¬* autotune]
Lemma-based	[ekf]	[radio failsafe]	[battery failsafe]
POS-based	[copter], [*¬*autotune ∧ copter]	[rtl], [copter]	[battery failsafe]
Root-based	[copter]	[*¬* autotune], [*¬*ekf]	[*¬* autotune], [*¬* ekf]

According to Table 3, the “copter” can be attributed to the type of the UAV (i.e., multicopter that generates lift using rotating blades instead of fixed wings). “Copter” was chosen correctly as one of the actual causes by HP2SAT using the baseline, POS-based, and root-based graphs. The “ekf” (extended kalman filter) error occurred after crashing to the ground and hence is not a correct diagnose. Thus, using the graphs from baseline and lemma-based techniques resulted in partially incorrect diagnoses. On the other hand, POS-based and root-based graphs led to the correct diagnoses. Using the POS-based graph resulted in a more comprehensive diagnosis by detecting the untuned controller parameters (i.e., *¬*autotune).

#### 5.4.2 Category 2: events during takeoff and in cruise

Flight logs 4 and 5 belong to this category. Due to space constraints, we provide a summary of the diagnoses in the second column of Table 3, while elaborating only on flight log 4 in detail here. The altitude signal of the UAV during flight log 4 is depicted in Figure 12. In this flight, the UAV flew to over 100 m and lost its radio connection. As a result, the radio failsafe triggered the “return to launch” (RTL) maneuver. This Manuever caused the “input throttle” to fall to zero. Consequently, the UAV which had a quadcopter structure, lost its balance and fell to the ground.

According to Table 3, “Radio failsafe”, “copter”, and “rtl” are plausible diagnoses provided by the baseline, lemma-based, and POS-based methods. However, using the root-based graph results in irrelevant diagnoses in our point of view. *¬*autotune (no autotune) is irrelevant because even doing an autotune could not enable system to stand the zero throttle conditions. *¬*ekf (no extended kalman filter) suggests that non-occurrence of the perception error is an actual cause, which is not valid.

#### 5.4.3 Category 3: equipment problems

Flight logs 6 to 8 belong to this category. Due to space constraints, we provide a summary of the diagnoses in the third column of Table 3, while elaborating only on flight log 6 in detail here. The altitude signal of the UAV during the last 2 minutes of flight log 6 is depicted in Figure 12. In this flight, battery was drained out after around 13 min of flight and the UAV started to lose altitude. Contrary to its original purpose, the “battery failsafe” contributed to the problem by causing the UAV to fly away from its starting point. Had the “battery failsafe” procedure immediately control the horizontal position, the crash would not have occurred. Note that the “ekf” error that occurred after crashing to the ground is not an actual cause of the occurred crash, but an effect of the hitting the ground.

According to Table 3, the root-based graph results in wrong diagnoses because its raw version only had “battery” instead of “battery failsafe.” The node “battery” was dropped in the node filtering process, because it was too generic to be mapped to the monitored events. The baseline graph fails, because although, its raw version has a “battery failsafe,” there are no paths between it and the *crash* event and hence, this node was removed from the final baseline graph. This example demonstrates that HP2SAT may generate erratic diagnoses, if the provided causal graph does not contain relevant nodes or edges.

**RQ5**: We find that the POS-based graph shows a superior diagnosis validity compared to the rest of the graphs in diagnosing the flight logs in our ArduCrash dataset.

### 5.5 Discussion

The scraping technologies are effective and mature enough in gathering millions of sentences from the Internet. Although our cause–effect extraction module achieves better precision and recall than a state-of-the-art deep-learning based tool (i.e., CiRA), its performance is still lower than the human level. The merging and filtering techniques based on the observability and relevance of the nodes heavily affect the generated graphs and hence, the diagnoses computed based on them. The POS-based graph results in better diagnoses in our ArduCrash dataset. One reason could be that it provides a trade-off between the abstraction and inclusiveness of the nodes. In any case, an extensive analysis on more flight logs is required to have a conclusive comparison among the merging techniques.

### 5.6 Threats to validity

#### 5.6.1 Internal threats

Our cause–effect phrase labels may differ from Ardupilot experts’ opinions. To reduce the subjectivity of the annotations, two authors of this paper with different levels of expertise in the UAV domain annotated the 539 sentences of our ground truth. Next, the performance of the extraction was computed against the two sets of annotation and the average was taken as the final performance.

The technical validity of statements in the online resources of Ardupilot should be taken with a grain of salt, because there is no qualification procedure for participating in discussion fora, Discord chats, or even writing user manuals. To increase the confidence level in the extracted cause–effect phrases, we could drop the causal pairs with too few mentions in our corpora. However, in our current implementations, this could significantly reduce the number of identified cause-effect pairs. In general, the confidence level in the causal statements of the users needs to be taken into account for better results.

#### 5.6.2 External threats

Our extraction module achieves 65% mean precision and 42% mean recall on our ground truth dataset, which is lower than human-level performance. Nonetheless, it serves our goal to demonstrate how the cause–effect extraction module can be used in the diagnosis pipeline. In case more accurate extraction algorithms are proposed in the literature, integrating them into our framework is straightforward.

Our primary use case in this study is the Ardupilot UAV controller, which is only one instance of the open-source autopilot systems. Nevertheless, Ardupilot is the most popular open-source UAV controller according to its project statistics on Github^22^. Moreover, it is designed on the basis of the *sense-plan-act* pattern, which is a standard architecture for autonomous systems.

Another issue about our internal use case is that cause–effect phrases from Ardupilot web resources may contain information about various types of UAVs such as multi-rotors, fixed-wing airplanes, and helicopters. In any case, we expect to have mostly non-conflicting knowledge for the three types because physical laws and many software components are the same for all of them. Hence, we see Ardupilot (and its primary UAV type, which is quadcopter) as a suitable case study for identifying and addressing the challenges of NLP-based diagnosis in UAVs.

## 6 Conclusion

This study proposes a novel methodology to utilize powerful NLP tools to generate causal graphs for UAV diagnosis. We identified four main challenges in realizing an end-to-end solution, among which cause–effect extraction is the most crucial one. We combined several cause–effect extraction techniques to realize a multi-token and multi-pair extraction module. Moreover, we demonstrated how different node merging techniques affect the structure of the generated causal graphs. Finally, we demonstrated how the graphs could be used to diagnose a real UAV failure based on HP’s definition of ACA. In summary, the results indicate the feasibility of gathering causal knowledge from textual data and using it to diagnose real UAV failures. Although, not all of our generated diagnoses are correct, our proposed methodology is a promising approach in improving the safety of UAVs by automating the failure diagnosis process.

Several directions can be followed up in the future. First, better preprocessing can result in more and cleaner sentences in the main corpus. By analyzing sentences with pronouns and tracing them back to find the referred concept, we will be able to extract more usable cause–effect pairs. Approximately 935K raw sentences were collected, which could be increased by scraping more web pages including generic UAV websites. In any case, there should be a trade-off between the comprehensiveness and specificity of the gathered information. Moreover, the NLP models we use to tokenize and tag the sentences are already trained on generic datasets including 300K web data and 120K telephone conversations (Weischedel et al., 2013). Language models tuned on a corpora from the study context (i.e., articles related to UAVs) may lead to better results.

Second, pure machine learning techniques, rather than our hybrid approach, can be investigated. Although our hybrid extraction method performed better than CiRA, building proper rules to extract cause–effect phrases is time-consuming. In general, data-driven methods are more scalable and can automate the entire process, even though they rely on the availability of large labeled data. Moreover, the confidence in the extracted cause–effect pairs can be increased by considering the experience of the users that mentioned them or by assigning a confidence value on the basis of the frequency of discovered causal relationships.

Third, different approaches can be applied to equate cause–effect phrases. An advanced word embedding mechanism trained on the system context is expected to result in a more effective node merging.

Finally, the correctness and completeness of other abductive reasoning algorithms, such as consistency-based diagnosis (Peischl and Wotawa, 2003), should be compared with our implementation of the ACA procedure. A qualitative study on user satisfaction is of interest to determine whether identifying the actual causes results in preventing future failures of the system.

## Data Availability

The datasets presented in this study can be found in online repositories. The names of the repository/repositories and accession number(s) can be found below: https://doi.org/10.6084/m9.figshare.21711608.

## Footnotes

1
https://ardupilot.org/copter/index.html

2
https://ardupilot.org/copter/docs/common-downloading-and-analyzing-data-logs-in-mission-planner.html

3
https://discuss.ardupilot.org/c/log-analysis-and-tuning/104

4
https://discuss.ardupilot.org/t/log-analysis-questions-about-expected-behavior-on-compass-failure/47031

5
https://discuss.ardupilot.org/t/setting-the-maximum-descent-rate-in-apm/2210/7

6 Actual causation has several definitions in the literature, which we discuss in Section 2.4.

7
http://www.fminer.com/

8
https://scrapy.org/

9
https://www.selenium.dev/

10
https://docs.scrapy.org/en/latest/topics/spiders.html

11
https://selenium-python.readthedocs.io/

12
https://spacy.io/

13 We observe that most negated sentences in ArduCorpus imply that the user was not sure about the statement. However, some useful sentences, which discuss the non-occurrence of events, may also be eliminated by this filter. In any case, there should be a trade-off between the quality and the quantity of the sentences.

14 Activate, actuate, arouse, associate, cause, commence, derive, effect, effectuate, elicit, entail, evoke, generate, implicate, induce, kindle, lead, link, produce, provoke, relate, result, spark, stem, stimulate, trigger, unleash.

15 We investigate the proper level of the phrase abstraction in the graph building module.

16 These tags serve our purpose: csubjpass, nsubjpass, nsubj, csubj, dobj, pobj, nn, amod, nmod, advmod, compound, prep, poss.

17
https://ardupilot.org/dev/index.html

18
https://github.com/PX4/PX4-Autopilot

19
https://github.com/paparazzi/paparazzi

20
https://www.act.nato.int/futures-work

21
https://ardupilot.org/copter/docs/crash_check.html

22
https://github.com/ArduPilot/ardupilot

## References

[B1] Ackerman E. Strickland E. (2018). Medical delivery drones take flight in east africa. IEEE Spectr. 55, 34–35. 10.1109/mspec.2018.8241731

[B2] Ahne A. Khetan V. Tannier X. Rizvi M. I. H. Czernichow T. Orchard F. (2022). Extraction of explicit and implicit cause-effect relationships in patient-reported diabetes-related tweets from 2017 to 2021: deep learning approach. JMIR Med. Inf. 10, e37201. 10.2196/37201 PMC9346561 35852829

[B3] Baidya S. Shaikh Z. Levorato M. (2018). “Flynetsim: an open source synchronized uav network simulator based on ns-3 and ardupilot,” in Proceedings of the 21st ACM International Conference on Modeling, Analysis and Simulation of Wireless and Mobile Systems, 37–45. 10.1145/3242102.3242118

[B4] Baldi S. Sun D. Xia X. Zhou G. Liu D. (2022). Ardupilot-based adaptive autopilot: architecture and software-in-the-loop experiments. IEEE Trans. Aerosp. Electron. Syst. 58, 4473–4485. 10.1109/taes.2022.3162179

[B5] Chaulagain R. S. Pandey S. Basnet S. R. Shakya S. (2017). “Cloud based web scraping for big data applications,” in 2017 IEEE International Conference on Smart Cloud (SmartCloud) (IEEE), 138–143.

[B6] Choi J. D. Palmer M. (2012). Guidelines for the clear style constituent to dependency conversion. Technical Report, 1. Center for Computational Language and Education Research, University of Colorado Boulder, Institute of Cognitive Science, 12.

[B7] Coleman T. F. Moré J. J. (1983). Estimation of sparse jacobian matrices and graph coloring blems. SIAM J. Numer. Analysis 20, 187–209. 10.1137/0720013

[B8] De Marneffe M.-C. Manning C. D. (2008). “The stanford typed dependencies representation,” in Coling 2008: proceedings of the workshop on cross-framework and cross-domain parser evaluation, 1–8.

[B9] Doan S. Yang E. W. Tilak S. S. Li P. W. Zisook D. S. Torii M. (2019). Extracting health-related causality from twitter messages using natural language processing. BMC Med. Inf. Decis. Mak. 19, 79–77. 10.1186/s12911-019-0785-0 PMC6448183 30943954

[B10] Fischbach J. Frattini J. Spaans A. Kummeth M. Vogelsang A. Mendez D. (2021). “Automatic detection of causality in requirement artifacts: the cira approach,” in International Working Conference on Requirements Engineering: Foundation for Software Quality (Springer), 19–36.

[B11] Frattini J. Junker M. Unterkalmsteiner M. Mendez D. (2020). “Automatic extraction of cause-effect-relations from requirements artifacts,” in Proceedings of the 35th IEEE/ACM International Conference on Automated Software Engineering, 561–572.

[B12] Gentzel A. Garant D. Jensen D. (2019). The case for evaluating causal models using interventional measures and empirical data. Adv. Neural Inf. Process. Syst. 32.

[B13] Girju R. Moldovan D. I. (2002). Text mining for causal relations. FLAIRS Conf., 360–364.

[B14] Haddaway N. (2015). The use of web-scraping software in searching for grey literature. Grey J. 11, 186–190.

[B15] Halpern J. (2015). “A modification of the halpern-pearl definition of causality,” in Twenty-Fourth International Joint Conference on Artificial Intelligence.

[B16] Hassanzadeh O. Bhattacharjya D. Feblowitz M. Srinivas K. Perrone M. Sohrabi S. (2019a). “Answering binary causal questions through large-scale text mining: an evaluation using cause-effect pairs from human experts,” in Proceedings of the Twenty-Eighth International Joint Conference on Artificial Intelligence, Macao, China, August 10-16, 2019 (IJCAI).

[B17] Hassanzadeh O. Bhattacharjya D. Feblowitz M. Srinivas K. Perrone M. Sohrabi S. (2019b). Data sets of cause-effect pairs. 10.5281/zenodo.3214925

[B18] Ibrahim A. Kacianka S. Pretschner A. Hartsell C. Karsai G. (2019a). “Practical causal models for cyber-physical systems,” in NASA Formal Methods Symposium (Springer), 211–227.

[B19] Ibrahim A. Pretschner A. (2020). “From checking to inference: actual causality computations as optimization problems,” in International Symposium on Automated Technology for Verification and Analysis (Springer), 343–359.

[B20] Ibrahim A. Rehwald S. Pretschner A. (2019b). Efficient checking of actual causality with sat solving. Eng. Secure Dependable Softw. Syst. 53, 241.

[B21] Kayesh H. Islam M. S. Wang J. Anirban S. Kayes A. Watters P. (2020). “Answering binary causal questions: a transfer learning based approach,” in 2020 International Joint Conference on Neural Networks (IJCNN) (IEEE), 1–9.

[B22] Khurana D. Koli A. Khatter K. Singh S. (2022). Natural language processing: state of the art, current trends and challenges. Multimedia Tools Appl. 82, 3713–3744. 10.1007/s11042-022-13428-4 PMC9281254 35855771

[B23] Kueffner K. R. (2021). A comprehensive survey of the actual causality literature. 10.34726/hss.2021.90003

[B24] Kutuzov A. Fares M. Oepen S. Velldal E. (2017). “Word vectors, reuse, and replicability: towards a community repository of large-text resources,” in Proceedings of the 58th Conference on Simulation and Modelling (Linköping University Electronic Press), 271–276.

[B25] Landers R. N. Brusso R. C. Cavanaugh K. J. Collmus A. B. (2016). A primer on theory-driven web scraping: automatic extraction of big data from the internet for use in psychological research. Psychol. methods 21, 475–492. 10.1037/met0000081 27213980

[B26] Luo Z. Xiang X. Zhang Q. (2019). “Autopilot system of remotely operated vehicle based on ardupilot,” in International Conference on Intelligent Robotics and Applications (Springer), 206–217.

[B27] Maisonnave M. Delbianco F. Tohme F. Milios E. Maguitman A. G. (2022). Causal graph extraction from news: a comparative study of time-series causality learning techniques. PeerJ Comput. Sci. 8, e1066. 10.7717/peerj-cs.1066 PMC9374167 35967930

[B28] Melchor R. A. Fonseca M. Rey B. Hernandez A. Puertas B. Gomez S. (2020). Ct-152: application of web-scraping techniques for autonomous massive retrieval of hematologic patients’ information during sars-cov2 pandemic. Clin. Lymphoma Myeloma Leukemia 20, S214. 10.1016/s2152-2650(20)30778-3

[B29] Mitchell R. (2018). Web scraping with Python: collecting more data from the modern web. Sebastopol, California: “O’Reilly Media, Inc.”.

[B30] Morgan S. L. Winship C. (2014). Analytical methods for social research. 2 edn. Cambridge University Press, 77–102. Causal Graphs. 10.1017/CBO9781107587991.004

[B31] Pearl J. (1998). Graphical models for probabilistic and causal reasoning. Quantified Represent. Uncertain. imprecision, 367–389. 10.1007/978-94-017-1735-9_12

[B32] Peischl B. Wotawa F. (2003). Model-based diagnosis or reasoning from first principles. IEEE Intell. Syst. 18, 32–37. 10.1109/mis.2003.1200725

[B33] Rajput A. E. Ahmed S. M. (2019). Big data and social/medical sciences: state of the art and future trends. *arXiv preprint arXiv:1902.00705* .

[B34] Sharp R. Surdeanu M. Jansen P. Clark P. Hammond M. (2016). Creating causal embeddings for question answering with minimal supervision. *arXiv preprint arXiv:1609.08097* .

[B35] Sorgente A. Vettigli G. Mele F. (2013). Automatic extraction of cause-effect relations in natural language text. DART@ AI* IA 2013, 37–48.

[B36] Torresan C. Berton A. Carotenuto F. Di Gennaro S. F. Gioli B. Matese A. (2017). Forestry applications of uavs in europe: a review. Int. J. Remote Sens. 38, 2427–2447. 10.1080/01431161.2016.1252477

[B37] Weischedel R. Palmer M. Marcus M. Hovy E. Pradhan S. Ramshaw L. (2013). Ontonotes release 5.0 ldc2013t19. Philadelphia, PA 23: Linguistic Data Consortium.

[B38] Wild G. Murray J. Baxter G. (2016). Exploring civil drone accidents and incidents to help prevent potential air disasters. Aerospace 3, 22. 10.3390/aerospace3030022

[B39] Yang J. Han S. C. Poon J. (2021). A survey on extraction of causal relations from natural language text. *arXiv preprint arXiv:2101.06426* .

[B40] Zibaei E. Banescu S. Pretschner A. (2018). Diagnosis of safety incidents for cyber-physical systems: a uav example. In 2018 3rd International Conference on System Reliability and Safety (ICSRS) (IEEE), 120–129.

